# A Subset of Patients With Autism Spectrum Disorders Show a Distinctive Metabolic Profile by Dried Blood Spot Analyses

**DOI:** 10.3389/fpsyt.2018.00636

**Published:** 2018-12-07

**Authors:** Rita Barone, Salvatore Alaimo, Marianna Messina, Alfredo Pulvirenti, Jean Bastin, MIMIC-Autism Group, Agata Fiumara, Alfredo Ferro, Richard E. Frye, Renata Rizzo

**Affiliations:** Referral Centre for Inherited Metabolic Disorders, Department of Clinical and Experimental Medicine, University of Catania, Catania, Italy; Child Neurology and Psychiatry, Department of Clinical and Experimental Medicine, University of Catania, Catania, Italy; Pediatric Hematology, Department of Clinical and Experimental Medicine, University of Catania, Catania, Italy; Institute of Biostructures and Bioimaging (IBB), National Research Council (CNR), Catania, Italy; ^1^Child Neurology and Psychiatry, Department of Clinical and Experimental Medicine, University of Catania, Catania, Italy; ^2^Referral Centre for Inherited Metabolic Disorders, Department of Clinical and Experimental Medicine, University of Catania, Catania, Italy; ^3^Bioinformatics Unit, Department of Clinical and Experimental Medicine, University of Catania, Catania, Italy; ^4^Sorbonne Paris Cité, Faculté des Sciences Fondamentales et Biomédicales, Université Paris Descartes, Paris, France; ^5^INSERM, UMR-S 1124, Toxicologie, Pharmacologie et Signalisation Cellulaire, Paris, France; ^6^University of Arizona College of Medicine, Phoenix, AZ, United States; ^7^Phoenix Children's Hospital, Phoenix, AZ, United States

**Keywords:** autism spectrum disorders, dried blood spots, ESI-MS/MS, mitochondrial fatty acid β-oxidation, machine learning

## Abstract

Autism spectrum disorder (ASD) is currently diagnosed according to behavioral criteria. Biomarkers that identify children with ASD could lead to more accurate and early diagnosis. ASD is a complex disorder with multifactorial and heterogeneous etiology supporting recognition of biomarkers that identify patient subsets. We investigated an easily testable blood metabolic profile associated with ASD diagnosis using high throughput analyses of samples extracted from dried blood spots (DBS). A targeted panel of 45 ASD analytes including acyl-carnitines and amino acids extracted from DBS was examined in 83 children with ASD (60 males; age 6.06 ± 3.58, range: 2–10 years) and 79 matched, neurotypical (NT) control children (57 males; age 6.8 ± 4.11 years, range 2.5–11 years). Based on their chronological ages, participants were divided in two groups: younger or older than 5 years. Two-sided *T*-tests were used to identify significant differences in measured metabolite levels between groups. Näive Bayes algorithm trained on the identified metabolites was used to profile children with ASD vs. NT controls. Of the 45 analyzed metabolites, nine (20%) were significantly increased in ASD patients including the amino acid citrulline and acyl-carnitines C2, C4DC/C5OH, C10, C12, C14:2, C16, C16:1, C18:1 (*P*: < 0.001). Näive Bayes algorithm using acyl-carnitine metabolites which were identified as significantly abnormal showed the highest performances for classifying ASD in children younger than 5 years (n: 42; mean age 3.26 ± 0.89) with 72.3% sensitivity (95% CI: 71.3;73.9), 72.1% specificity (95% CI: 71.2;72.9) and a diagnostic odds ratio 11.25 (95% CI: 9.47;17.7). Re-test analyses as a measure of validity showed an accuracy of 73% in children with ASD aged ≤ 5 years. This easily testable, non-invasive profile in DBS may support recognition of metabolic ASD individuals aged ≤ 5 years and represents a potential complementary tool to improve diagnosis at earlier stages of ASD development.

## Introduction

Autism spectrum disorder (ASD) is a neurodevelopmental disorder that affects approximately 16.8 per 1,000 (one in 59) children aged 8 years in the US and with a male/female ratio of 4:1 (1). In Italy, a recent study reported an overall prevalence rate of one in 100 children at age 7–8 years (2). ASD is characterized by significant defects of social communication and interaction and by restricted and repetitive patterns of interests and activities with onset in early childhood (3). The etiology remains poorly understood. Increasing evidence has converged on possible interactions among pleiotropic genetic background conferring vulnerabilities to environmental inputs leading to multiple systemic co-morbidities including metabolic disarrangement (4). Classic inborn errors of metabolism (IEM) affect a subgroup of ASD patients accounting for 1–3% of patients (5). Acquired symptoms featuring autism or childhood disintegrative disorder may occur in the neuronopathic lysosomal storage disorders (6, 7). Among IEM, primary mitochondrial diseases affect nearly 5% of patients with ASD, however the occurrence of abnormal biomarkers indicating mitochondrial dysfunction is higher in patients with ASD than in the general population (8). On a clinical ground, children with ASD may exhibit features of a mitochondrial disease such as hypotonia and delayed motor development as well as gastrointestinal disturbances and regression following fever or other environmental triggers (9).

Clinical diagnosis of ASD relies on behavioral tests. Early recognition and specialized intervention improve the outcome and are most effective if initiated early in life (10). Thus, the development of multiple laboratory markers that can assist in the early and accurate diagnosis of ASD is envisaged. Urinary metabolomic studies (11–16) and a few studies performed on blood samples (17–19) collectively showed modification of amino acid, purine and fatty acid metabolic pathways, increased oxidative stress, gut dysbiosis and altered gut permeability in individuals with ASD. Multiplatform analytical methodology and multivariate analysis may provide the best models discriminating between ASD and typically developing (TD) children. Through this approach, a rigorous analysis for the discovery of ASD biomarkers combined several mass spectrometry (MS)-based analyses of blood. This combined analysis resulted in 40 features could differential ASD and TD samples with an accuracy of 70% (17). More recently, a study in 38 children with ASD reported increased advanced glycation endproducts, Nε-carboxymethyllysine and Nω-carboxymethylarginine, and increased oxidation damage marker, dityrosine, in plasma proteins, capable to classify the disease status (19).

Since the 1980s, electrospray ionization (ESI) and tandem MS/MS technology endorsed high throughput analyses of samples extracted from dried blood spots (DBS) for newborn screening of IEM as health care standard (20). Thus, we hypothesized that ESI-MS/MS analyses of different metabolites in DBS might represent a high throughput method for metabolic profiling of individuals with ASD by a single injection, in a rapid, low-cost, and suitable procedure. To test this hypothesis, we used a standardized ESI-MS/MS analyses in DBS to systematically examine the levels of a large panel of highly selective biochemical analytes in patients with ASD and healthy TD, matched-control subjects. The targeted metabolites include acyl-carnitines and amino acids representing a set of ASD candidate metabolic markers. We propose a novel approach applying machine learning methods to assess differences in the metabolic profile between ASD and age-matched healthy TD controls. This represents a promising novelty in the field given that previous analyses of multiple analytes in ASD often resort to a one-at-a-time approach that does not consider the data as a whole. Using univariate and multivariate data modeling, we outlined a metabolic risk profile capable to classify a subset of ASD patients from TD children. The study supports identification of metabolic ASD subtype whose distinguishing features suggest a reduced flux through the mitochondrial fatty acid β-oxidation (FAO) pathway.

## Methods

A total of 162 Caucasian subjects with age ranging from 30 months to 11 years were included in a case-control study during an 18-month period (January 1, 2016–June 30, 2017) at the Child Neurology and Psychiatry Unit of the University Children Hospital Catania, Italy. Participants comprised 83 children with the ASD diagnosis (60 males, 23 females; age 6.06 ± 3.6; range: 2–10 years) and 79 healthy TD controls, with a similar age and gender distribution as the patients (57 males, 22 females; age 6.8 ± 4.1; range 2.5–11 years) (Table 1). The Institutional Review Board at University Hospital of Catania approved the study that was performed in accordance with the ethical standards laid down in the 1964 Declaration of Helsinki and its later amendments (Helsinki Declaration 1975, revision 2013). Written informed consent was obtained from all ASD participants' parent or legal guardian in order to enter clinical and laboratory data from the clinical files into the present study. Diagnosis of ASD was obtained according to strict criteria using standardized diagnostic tests including the Autism Diagnostic Interview-Revised (ADI-R) (21) and Autism Diagnostic Observation Schedule (ADOS) (22). The Calibrated Severity Score (CSS) from 4 to 10 was used as a measure of autism severity (23). Developmental quotient (DQ) and/or Intellectual quotient (IQ) were measured in all participants by a comprehensive, standardized neuropsychological assessment battery administered according to age. Among ASD individuals, exclusion criteria were the presence of an associated monogenic disease (i.e., Fragile-X syndrome, Tuberous Sclerosis), positive chromosomal microarray analysis, positive history for mitochondrial disease or known medical conditions including autoimmune disease and inflammatory bowel diseases (IBD)/celiac disease.

**Table 1:** Demographic and clinical characteristics of ASD patients and TD controls divided by age.

**ASD**					**TD**			
**Participant characteristics**	**Total sample (*n* = 83)**	**Age ≤ 5 (*n* = 42)**	**Age > 5 (*n* = 41)**	*P*-value*	**Total sample (*n* = 79)**	**Age ≤ 5 (*n* = 35)**	**Age > 5 (*n* = 44)**	*P-*value^*^
Age (years)	6.06 ± 3.58	3.26 ± 0.89	8.9 ± 2.98	n.a.	6.8 ± 4.11	3.06 ± 1.5	9.7 ± 2.86	n.a.
Boys (%)	60 (72.3)	29 (69.04)	31 (75.6)	0.653	57 (72.1%)	24 (68.5%)	33(75%)	0.692
DQ/IQ	63.2 ± 20.8	56.8 ± 17.1	69.6 ± 22.4	0.022	93.9 ± 14.2	89.6 ± 11.2	95.3 ± 10.5	0.752
DD/ID (%)	54 (65.1)	32 (76.2)	22 (53.6)	0.035	n.a	n.a.	n.a.	n.a.
Regression (%)	29 (35)	19 (45.2)	10 (24.4)	0.065	n.a	n.a.	n.a.	n.a.
Autism severity (ADOS CSS)°	6.7 ± 1.8	6.6 ± 1.8	6.8 ± 1.8	0.845	n.a.	n.a	n.a.	n.a.

* *Fisher's Exact Test was performed for discrete variables gender, DD/ID and regression. T-test was performed for continuous variables DQ/IQ and ADOS-CSS. °The Social Communication Questionnaire was used to screen and exclude autism in TD children. DQ, developmental quotient; IQ, intelligence quotient; DD, developmental disability; ID, intellectual disability; ADOS, Autism Diagnostic Observation Schedule. CSS, Calibrated Severity Score. n.a., not applicable*.

TD children were recruited among subjects that underwent morning fasting blood analyses screening for sideropenic anemia that was definitely ruled out in all included TD participants. Full informed consent was signed from parents to participate in the study. TD participants' exclusion criteria included positive history for inherited metabolic diseases, intellectual disability or other developmental, neurological, or behavioral problems and inflammatory bowel diseases/celiac disease. The Social Communication Questionnaire (24) was used to screen and exclude autism in TD children. Since artifacts in plasma acylcarnitine levels are possible due to diet enriched with fatty acids (MCT-oil, ketogenic diet) (25), we ensured that no participants underwent fatty acids enriched diet, such as ketogenic diet or MCT-oil, at least 6 months before sample collection.

### Metabolic Work-Up in ASD Subjects

ASD patients underwent blood and urine collection in the morning between 8.00 and 8.30 a.m. after nocturnal fasting. Routine blood analyses including glucose, transaminases, cholesterol, triglycerides, creatine kinase, electrolytes and thyroid hormones were normal. Morning fasting lactate and ammonia blood levels were increased in 12.5 and 22.2% of patients, respectively in line with previous reported rates of increased markers of mitochondrial dysfunction in ASD (8). Twenty-five out of 40 studied subjects (62.5%) had significantly decreased blood Vitamin D3 levels with normal Ca/P ratio. Urinary organic acids by using Gas Chromatography/MS detected increased excretion of ketone bodies in five patients. One patient showed increased urinary 3-hydroxy-isovaleric acid with normal plasma biotinidase activity. In two sibs with ASD, the acylcarnitine profile showed increase of C8, C10, C10:1 carnitine levels suggesting medium-chain acyl-CoA dehydrogenase deficiency (MCAD). Molecular analyses was not significant for any mutations associated to MCAD in these patients.

### Biospecimen Collection, Processing and MS/MS Analysis

To avoid systematic differences related to the time of sample collection, blood spots on filter paper card (Whatman card Specimen 903) were collected from each participant in the ASD and TD groups in the morning between 8.00 and 8.30 a.m. after nocturnal fasting. Samples from the NT children were prospectively collected in the same period, along with ASD children samples. Once dried, blood spots were stored at 4°C in a unique refrigerator with controlled humidity rate and processed within 2 weeks after sampling.

A 3.2 mm diameter blood dot of each individual was used for the analyses. Underivatized specimens were analyzed using electrospray ionization (ESI)-Tandem MS/MS system. Forty-five metabolites including amino acids, free carnitine and acyl-carnitines (saturated, unsaturated, hydroxylated, and dicarboxylated) were simultaneously measured in DBS. The analyte concentration was quantified by comparison with known concentration of corresponding stable-isotope internal standards. Results of targeted 45 metabolites in ASD participants were considered in comparison with age-matched reference ranges obtained from studied TD healthy subjects.

### Statistical Analyses

Blood levels of forty-five targeted analytes (μmol/L) obtained from 162 subjects, 83 ASD patients and 79 TD healthy controls, were evaluated. Metabolites, with statistically significant different blood levels between ASD and healthy TD control children were identified by using the R package limma (26). Since data supports equal population variances together with normal distributions, a *T*-test was applied. The *p*-value produced by the two-sided *T*-test, employed by limma, was corrected using the Benjamini & Hochberg method in order to estimate the False Discovery Rate (27). All differences were considered to be statistically significant at a 5% probability level. Possible associations between the identified metabolites and clinical features of ASD patients were verified by Spearman correlations analyses.

### Classification Modeling

Model development was performed with the aim to detect metabolic features useful to profile ASD patients vs. healthy NT controls. For this purpose we trained an algorithm on the discriminant metabolites identified as described. The methodology was evaluated in terms of Sensitivity = TP/(TP + FN), Specificity = TN/(TN + FP), where TP is the number of true positives, i.e., the number of patients correctly classified; TN is the number of true negatives, i.e., number of controls correctly classified; FP is the number of false positives, i.e., number of controls classified as patients; and FN is the number of false negatives, i.e., number of patients classified as controls. Diagnostic odds ratios and 95% confidence intervals were evaluated.

**Figure 1:**
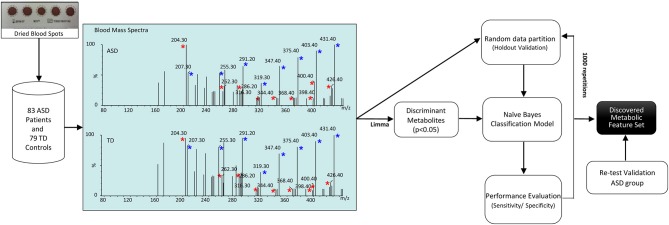
Workflow of the study. Blood acylcarnitines (m/z) C2 (204.3); C4DC\C5OH (262.2); C10 (316.2); C12 (344.3); C14:2 (368.3); C16 (400.3); C16:1 (398.3); C18:1 (286.2) (red asterisks) measured in DBS are increased in patients with ASD. Näive Bayes algorithm trained on the identified metabolites was used to profile children with ASD vs. healthy controls. Blue asterisks indicate correspondent stable-isotope internal standards.

The workflow of the study is depicted in Figure 1. Our dataset, comprising 83 ASD patients and 79 healthy controls, was randomly partitioned into a training set of 124 samples (67 ASD patients and 57 healthy controls) for identification of the classification modeling, and 38-sample holdout set (16 ASD patients and 22 healthy controls). Due to the small cohort size, keeping a large part of the samples in the training set is needed to proper identify the classification model (28). For this reason, we kept two third of the samples in the training set and the remainder was used as a holdout validation set. Samples were properly randomized using diagnosis, age and gender to establish a similar proportion of factors on both training and holdout sets. Such holdout strategy was repeated 1,000 times to estimate average performances together with a 95% CI of the classification model. In addition, a validation test was performed in an independent set of 29 ASD participants randomly recruited for re-test analyses. Neurotypical controls were not included in this analysis because further blood sampling was not achieved in the TD group. Finally, classification performance was evaluated by permutation testing in order to establish a distribution of chance estimates. For this purpose, we trained the classifier with the 124-sample training set with randomized group labels (ASD vs. TD) many times (≈ 1,000). This allowed establishing a chance distribution that could be used for comparison.

## Results

### Participant Characteristics

Based on their chronological ages, participants were divided in two groups: younger (ASD n.42; TD n.35) or older than 5 years (ASD n.41; TD n.44). Demographic data and clinical features of all participants in the two age groups, such as presence of developmental delay (DD), intellectual disability (ID) (IQ < 70) and symptoms of regressive autism are presented in Table 1. The rate and extent of DD/ID were higher in children with ASD younger than 5 years (*P*: 0.035 and *P*: 0.0228, respectively). No significant differences were found in the rate of patients with regressive autism and in the degree of autism severity (CSS) between the two age groups (*P*: 0.065 and *P*: 0.845, respectively). ASD patients did not fulfill diagnostic criteria for probable or definite mitochondrial disorder according to Morava mitochondrial disease criteria system (29). Less than 5% of studied ASD children had hypotonia and/or epileptic seizures. None presented with ataxia, peripheral neuropathy, sensorineural deafness, cardiomyopathy or endocrinological problems which are common features of mitochondrial diseases.

### Metabolic Profile of Target Analytes by ESI-MS/MS of Blood Spots in Patients With ASD and Healthy Control Subjects

Over 45 analyzed metabolites in DBS, nine (20%) were significantly increased in ASD patients with respect to healthy, age-matched subjects (Table 2). The increased metabolites in ASD patients included eight acyl-carnitines such as short-chain (2-5 carbon length) C2 and C4DC\C5OH, medium-chain (6–12 carbon length) C10 and C12, and long-chain acyl-carnitines (13-18 carbon length) C14:2, C16, C16:1, C18:1. Among eleven studied amino acids, citrulline levels were increased in ASD patients (Table 2). Volcano plot showing the distribution of log-fold-changes vs. statistical significance (*p*-value) of the metabolites and individual swarm plots for the subject data, split by diagnosis, for the nine changed metabolites are reported in supplementary materials (Figures S1–S4). We estimated an effect size of 0.6 as the absolute difference between the mean of the most discriminant metabolites (short chain C2 and C4DC/C5OH acylcarnitines and long chain C10, C12, C14:2, C16, C16:1, C18:1 acylcarnitines, citrulline) within each class divided by the pooled variance observed between the two classes. This yielded a power of 0.93 at significance level of 0.05 suggesting a high practical significance. The power was also estimated within the two age groups at 0.79 for patient younger than 5 y. o. and 0.78 for older patients.

**Table 2:** Statistical significant metabolites in ASD participants with respect to TD participants.

**Metabolite**	**Abbreviation**	**Log-FC**	**Average concentration**				**t**	***p*-Value (ASD vs. TD)**	**Adjusted *p*-Value**
			**ASD**		**TD**				
			**≤5 y**	**>5 y**	**≤5 y**	**>5 y**			
Citrulline	CIT	0.3601	4.7594	4.5606	4.3556	4.2129	3.7337	0.0003	0.0020
Acetylcarnitine	C2	0.3318	3.5333	3.5503	3.1886	3.2330	4.2770	0.0000	0.0007
Methylmalonyl/3-OH- isovalerylcarnitine^*^	C4DC\C5OH	0.0762	0.4734	0.5062	0.4058	0.4224	4.4927	0.0000	0.0006
Decanoylcarnitine	C10	0.0395	0.1468	0.1643	0.1035	0.1352	2.6288	0.0004	0.0470
Dodecanoylcarnitine	C12	0.0246	0.0714	0.0762	0.0414	0.0556	4.1838	0.0000	0.0007
Tetradecadienoylcarnitine	C14:2	0.0109	0.0398	0.0377	0.0211	0.0322	3.5912	0.0004	0.0025
Hexadecanoylcarnitine	C16	0.1409	1.0594	1.0934	0.9499	0.9180	3.7904	0.0002	0.0019
Hexadecenoylcarnitine	C16:1	0.0145	0.0758	0.0805	0.0634	0.0637	3.6726	0.0003	0.0021
Octadecenoylcarnitine	C18:1	0.1302	1.0725	1.1304	0.9295	0.9945	3.8526	0.0002	0.0019

*Logarithm of the fold-change (Log-FC) between the classes, average concentration in each age subgroup, t-test statistic with its p-value and Benjamini & Hochberg adjusted p-value*.

* *Isomers or isobars metabolites. ASD, Autism Spectrum Disorders; TD, Typical Development; y, year*.

Spearman correlations showed that in the ASD sample metabolite levels did not correlate with age, developmental or intellectual quotient and autism severity score (CSS) (Table 3).

**Table 3:** Spearman correlations computed for the metabolites listed in Table 2 in relation to the quantitative clinical variables Age, DQ/IQ, and CSS.

	**Age**		**DQ/IQ**		**CSS**	
**Metabolite**		***p*-Value**		***p*-Value**		***p*-Value**
C5OH\C4DC^*^	0.0771	0.4939	0.1176	0.2959	−0.1122	0.3188
C2	−0.1497	0.1821	−0.0634	0.5741	−0.0083	0.9414
C12	0.1103	0.3271	−0.0415	0.7129	−0.1488	0.1850
C18:1	0.0093	0.9341	−0.0986	0.3814	0.0155	0.8906
C16	−0.0130	0.9084	−0.0380	0.7362	−0.1340	0.2328
CIT	−0.1751	0.1180	−0.0685	0.5434	−0.1230	0.2741
C16:1	0.1286	0.2526	−0.0818	0.4677	−0.0795	0.4803
C14:2	−0.0410	0.7163	0.0060	0.9578	−0.0842	0.4551
C10	0.0843	0.4545	−0.0649	0.5647	−0.0607	0.5906

*For each metabolite we report the computed correlation together with a p-value, which indicates whether the observed correlation is statistically significant, under the null hypothesis that values are uncorrelated*.

* *Isomers or isobars metabolites. DQ, developmental quotient; IQ, intelligence quotient; CSS, Calibrated Severity Score*.

### Training and Testing Set Model Performance

Next, we trained a classifier based on the Naïve Bayes algorithm making use of the training set and adopting as predictor variables only the nine metabolites differing significantly between ASD and TD subjects (*P*: < 0.001) (Table 2). The results were verified on the holdout set with the purpose of checking the robustness of the procedure.

To assess the model and the predictive power of the selected metabolites, we compared the Naïve Bayes algorithm, with other classification techniques such as C-tree, Random Forest (RF), Support Vector Machine (SVM), Linear Regression (LM), and Recursive Partition Tree (PART) (online methods). Our final choice fell on the Naïve Bayes algorithm due to its robustness and stability. The training procedure led to the selection of acyl-carnitines C2 and C4DC\C5OH, C10, C12, C14:2, C16, C16:1, C18:1 as the most promising classification variables. Naïve Bayes algorithm, using a 8 feature set, reaches an overall classification performance with 73.3% sensitivity (95% CI 72.6–73.9), 63.4% specificity (95% CI 62.8–64), 6.78 DOR (95% CI, 6.39–7.16).

### Predictive Performances of the Metabolic Profile for Participants Divided by Age

Taking into account that ASD are neurodevelopmental disorders we considered closely possible interactions of measured metabolites with participant ages. For this purpose, we divided the sample into two groups according to age (≤5 years and >5 years) and we applied the classifier to discriminate among ASD and TD control subjects in each age group. Table 4 presents the predictive performances of the metabolic profile (measures and 95% CI), using Naïve Bayes and other compared classifiers, for all participants; participants aged ≤ 5 years and >5 years.

**Table 4:** Classifiers performances for all participants **(A)**; participants aged ≤ 5 years **(B)** and >5 years **(C)**.

**Classifier**	**Sensitivity**	**Specificity**	**DOR**
**(A) All participants**			
Naïve Bayes	0.7332 [0.7267; 0.7397]	0.6345 [0.6287; 0.6404]	6.7823 [6.3956; 7.1690]
C-tree	0.6715 [0.6590; 0.6841]	0.4942 [0.4824; 0.5060]	2.7437 [2.6171; 2.8703]
RF	0.7296 [0.7229; 0.7364]	0.5670 [0.5608; 0.5731]	4.8079 [4.5668; 5.0490]
SVM	0.7319 [0.7250; 0.7388]	0.5939 [0.5875; 0.6003]	5.5428 [5.2650; 5.8207]
LM	0.6324 [0.6250; 0.6397]	0.6585 [0.6523; 0.6646]	4.4804 [4.2328; 4.7280]
PART	0.6281 [0.6196; 0.6365]	0.5657 [0.5576; 0.5738]	2.9980 [2.8474; 3.1486]
**(B) ≤age 5 years**			
Naïve Bayes	0.7237 [0.7137; 0.7337]	0.7209 [0.7125; 0.7293]	10.1235 [9.4725; 10.7746]
C-tree	0.7590 [0.7441; 0.7739]	0.3574 [0.3399; 0.3749]	2.8163 [2.6411; 2.9914]
RF	0.8270 [0.8187; 0.8353]	0.5464 [0.5358; 0.5569]	7.1375 [6.5862; 7.6888]
SVM	0.8017 [0.7925; 0.8109]	0.6202 [0.6105; 0.6300]	8.1844 [7.6207; 8.7481]
LM	0.6567 [0.6450; 0.6684]	0.6871 [0.6771; 0.6972]	7.1923 [6.6648; 7.7198]
PART	0.7031 [0.6914; 0.7149]	0.4532 [0.4407; 0.4658]	2.9037 [2.6924; 3.1150]
**(C) >age 5 years**			
Naïve Bayes	0.6757 [0.6665; 0.6849]	0.5692 [0.5610; 0.5775]	4.2905 [4.0193; 4.5616]
C-tree	0.5071 [0.4867; 0.5275]	0.5468 [0.5290; 0.5645]	1.6693 [1.5826; 1.7559]
RF	0.5881 [0.5780; 0.5982]	0.5248 [0.5157; 0.5339]	2.3622 [2.2069; 2.5175]
SVM	0.5878 [0.5780; 0.5976]	0.6295 [0.6208; 0.6382]	3.9369 [3.6500; 4.2238]
LM	0.6039 [0.5939; 0.6139]	0.6022 [0.5933; 0.6112]	3.7105 [3.4456; 3.9755]
PART	0.5264 [0.5143; 0.5386]	0.5367 [0.5260; 0.5474]	2.0030 [1.8714; 2.1347]

*Compared classification algorithms: Naïve Bayes, C-tree, Random Forest (RF), Support Vector Machine (SVM), Linear Regression Model (LM), and Recursive Partition Tree (PART). For each classifier sensitivity, specificity, of the model and diagnostic odds ratio (DOR) are shown. All the measures are reported together with the bounds of the 95% CI*.

We found an increased competitiveness of the framework for classifying ASD in toddlers (n: 42 subjects mean age 3.26 ± 0.89) 72.3% sensitivity (95% CI: 71.3;73.9), 72.1% specificity (95% CI: 71.2;72.9), and diagnostic odds ratio (DOR) 11.25 (95% CI: 9.47;17.74). Furthermore, by applying our classification framework to subjects older than 5 years of age, we found a reduction in performance compared to younger subjects: Sensitivity 67.5% (95% CI: 66.6; 68.4) Specificity 56.9% (56.1;57.7), DOR 4.29% (95% CI: 4.09;4.56).

### Validation Test

Results were confirmed on independent validation test. For validation analyses, a set of 29 ASD participants was randomly recruited for re-test analyses. For this purpose, blood spot collection for metabolite analyses was repeated at the same conditions a second time after a mean time interval of 6.86 ± 3.8 months. Data from re-test were used as validation set. The initial values from total 132 subjects were used as training set. The results show an overall accuracy of 69% (20 patients correctly classified as ASD, and 9 misclassified as healthy). Splitting the re-test set by age we found a greater accuracy in younger subjects (≤5 years of age) (*n* = 11 samples, 73% accuracy) compared to individuals older than 5 years (*n* = 18 samples, 67% accuracy). It should be noted that the re-test validation set only includes participants with an ASD diagnosis. Therefore, the validation can only test for true positive and false negative ASD classifications.

### Permutation Testing

Classifier performance was evaluated using permutation testing. Permutation testing can be used to evaluate the probability of getting specificity and sensitivity values higher than the ones obtained during the cross-validation procedure by chance. In order to establish a distribution of chance estimates, we trained the classifier with the 124-sample training set each time randomly assigning patient and control labels to each sample many times (≈ 1,000) and repeated the cross-validation procedure. The results show that the quality of the classification in such a case is even lower than expected values in the case of a random classifier model with 43.9% accuracy (95% CI: 43.3;44.5) (Table S1). We definitely demonstrated that the accuracy of the classifier (73%) is significantly better than expected by chance alone as the classification algorithms are actually able to extract molecular patterns that distinguish patients, with respect to a chance distribution.

## Discussion

ASD is a polygenic multifactorial disorder with variable underlying mechanisms including energy metabolism disarrangement among others (30). Recognition of specific classes of ASD patients by biological markers has been considering effective for better understanding molecular mechanisms and to guide tailored therapeutic strategies in patient subset (31). In the current study we pursued to set up an easily testable blood metabolic profile in DBS to support early recognition of metabolic subtype patients at risk for ASD diagnosis. We found in an ASD population without clinical relevant features secondary to primary mitochondrial disease, a significant increase of blood short-chain, long-chain acyl-carnitines and, to a lesser extent, medium-chain acyl-carnitines. Our findings in a Sicilian ASD population (Mediterranean area) confirm the same, unique pattern of acyl-carnitine profile, which has been first systematically detected in ASD individuals from US, (32) defining a broadest coverage of ethnic and regional groups.

It is worth noting that distinct metabolite differences could be related to co-morbid undiagnosed medical conditions such as gastrointestinal disturbances that are frequently observed in ASD. In the present study we found significantly increased citrulline levels in children with ASD. Citrulline is an intermediate metabolic amino acid produced primarily by enterocytes. Blood citrulline level is considered a biomarker of gastrointestinal mucosal surface and enterocyte integrity. Previous studies showed that citrulline levels are inversely correlated with severity of intestinal malabsorption disease (i.e., coeliac disease) and inflammatory bowel disease (IBD) such as Crohn's disease (33). Patients with classic citrullinemia (type I) (argininosuccinate synthetase 1 gene mutation) present with elevated citrulline levels along with hyperammonemia and variable neurological symptoms in the neonatal period or later on. Interestingly, it was demonstrated that cumulative exposure to ammonia and citrulline are the most reliable indicators of poorer cognitive functioning in patients with classic citrullinemia (34).

Moreover, the existence of distinct metabolite differences could relate to concurrent vitamin D deficiency that was observed in a large proportion of patients with ASD (62%) in this study. Vitamin D has a pivotal role in neurodevelopment through several mechanisms including gene regulation and anti-inflammation/immunological modulation. Lower Vitamin D levels were consistently reported in subsets of patients with ASD compared to healthy controls (35). Carnitine is mainly provided in the diet, but is synthesized at extremely low rates from trimethyl-lysine residues generated during protein catabolism and is excreted in the urine. In patients with nutritional rickets (vitamin D deficiency), an increased urinary excretion of carnitine may occur that is reversed by vitamin D supplementation (36). It may be argued that carnitine metabolism may be involved in patients with nutritional rickets. Possible links between vitamin D deficiency and carnitine deficiency should be further investigated also in view of the higher prevalence of both these conditions in patients with ASD.

As ASD is developmental in nature, we considered possible interactions of measured metabolites with participant ages. Profiles of carnitine and acyl-carnitines change significantly during the first year of life, but kept at the same level between 2 and 15 years (37). We split the sample in two age categories (< 5 y.o. and ≥ 5 y.o.) to understand possible predictive metabolic signatures capable of distinguishing ASD and TD individuals at early stages of ASD development. This threshold is consistent with reliable ASD diagnosis and effectiveness of early intervention. Indeed, the definite diagnosis of ASD is generally made between 3 and 5 years (38). Moreover, increasing evidences support the effectiveness of early interventions (behavioral, developmental and educational approaches) in pre-schoolers (aged 24–71 months) with ASD (39).

The results show higher classification performance (sensitivity 72.3%, specificity 72.1%) at younger ages and potential application to improve diagnosis at earlier stages of ASD development. Re-test analyses as a measure of validity in independent samples showed an accuracy (proportion of observations that were correctly classified into patient or control group) of 73% in children aged ≤ 5 years. It has to be noted that the validation set only includes participants with an ASD diagnosis and so the validation can test for true positive and false negative ASD classifications.

The present study confirms that patients with ASD may show a distinct metabolic profile, demonstrating that this can be used to identify a subset of ASD patients with respect to TD at younger ages. We verified that in each age group, clinical variables such as cognitive levels (DQ/IQ) and autism severity (CSS) did not correlate with the discriminant metabolite levels. This implies that both clinical features were irrelevant to clinically discriminate the identified patient subset. It would be interesting to further investigate if individual component of behavioral scores instead of global scores and/or additional neurological features might be more helpful at the clinical level using larger samples that allow patient stratification (31).

The predictive metabolic profile identified in the present study is strongly supported by significant biological and experimental data associated with ASD:

1. the present findings collectively suggest a reduced flux through the mitochondrial β-oxidation pathway in a subset of patients with ASD. The acyl-carnitine pattern found in ASD patients is not consistent with any known genetic disorders of fatty acid oxidation and organic acid metabolism, electron transport chain or urea cycle dysfunction, or other inherited metabolic diseases. Genetic defects of mitochondrial β-oxidation are a group of IEM caused by failure of a single mitochondrial enzyme of β-oxidation such as short chain acyl-CoA dehydrogenase (SCAD), medium chain acyl-CoA dehydrogenase (MCAD), very long chain acyl-CoA dehydrogenase (VLCAD) or long chain 3-hydroxyacyl-CoA dehydrogenase (LCHAD). Mitochondrial β-oxidation defects may be secondary to dysfunction of dependent processes, such as deficiencies of the carnitine fatty acid transporter system, or mitochondrial electron transfer flavoprotein system (multiple acyl-CoA dehydrogenase deficiency) (40). The occurrence of developmental delay, autistic-like behavior or ASD in genetic defects of mitochondrial β-oxidation (41) particularly VLCAD (42) and LCHAD (43) suggests that impaired mitochondrial β-oxidation may contribute to dysfunctional energetic metabolism in subsets of patients with ASD. Deletion of the TMLHE gene, which is the first step in carnitine synthesis pathway and located on the X chromosome, is found more often in males with non-dysmorphic autism suggesting that TMLHE deficiency is a risk factor for autism, albeit with low penetrance (estimated at 2–4%) (44). Children with ASD, as a group, are deficient in Carnitine (45) with this deficiency potentially related to gastrointestinal symptom (46). Additionally, supplementing with Carnitine has been shown to improve core symptoms of ASD in two double-blind placebo controlled studies (47, 48).
2. ASD features and ASD have been reported in patients with propionic acidemia (PA), a severe organic acidemia caused by propionic acid (PPA) accumulation due to propionyl-CoA carboxylase enzyme deficiency (49). Endogenous PPA derives from the catabolism of branched-chain amino acids and from odd-chain fatty acid catabolism. PPA is a fermentation product of many autism associated gut bacteria, and also a common food preservative (50). Intracerebral PPA injections in rodents induce behavioral, electrographic and biochemical changes consistent with rodent ASD model (PPA model) (51). Brain lipid analyses of PPA model show increase of short- and long-chain acyl-carnitines but not medium- chain acyl-carnitines (52). The acyl-carnitine profile of PPA model overlaps with those found in patients with ASD (32), also in the current study.
3. Dysregulated cortical layer formation and layer-specific neuronal differentiation demonstrated in the neocortex of children with ASD, suggest possible defects in cell-cycle processes as well as in cell fate specification (53). The carnitine palmitoyl transferase (CPT) system, which mediates the entry of long-chain fatty acids into the mitochondria for ß-oxidation, operates in astrocytes (54, 55) and in embryonic and adult neural stem cells (NSC) (56, 57). Recent evidences show that fatty acids might represent an important oxidative fuel during embryonic and early postnatal development and a reduced flux through the mitochondrial fatty acid β-oxidation impairs NSC self-renewal in the mammalian embryonic brain and potentiates their transition to lineage-restricted cells (IPCs) (54–56). As a whole, experimental findings show a pivotal role for mitochondrial fatty acid β-oxidation in controlling NSC-to-IPC transition in mammalian embryonic and adult brain, and propose NSC self-renewal as a cellular mechanism underlying the association between disturbances of mitochondrial fatty acid oxidation and autism (56, 57).

We found a combined acyl-carnitine pattern in patients with ASD indicative of impaired mitochondrial fatty acid β-oxidation. The identified acyl-carnitine profile is characterized by a pattern of more elevated acyl-carnitine species in comparison with age-matched reference ranges. The presence of short-, medium-, and more elevated long-chain acyl-carnitine species, might reflect a mild generalized defect in FAO capacities, such as in FAO electron shuttle protein ETF (electron transferring factor), which is involved in the transfer of electrons coming from the short-chain, medium-chain and long-chain acyl-CoA dehydrogenases isoforms to the respiratory chain. Electrons from ETF feed the respiratory chain at the level of ETFDH (ETF dehydrogenase), a respiratory chain enzyme which transfers these electrons to coenzyme Q. Both inborn ETF and inborn ETFDH deficiency have been described in human, associated to a variety of phenotypes (58). The mechanisms responsible for expression of abnormal acyl-carnitine pattern in this subset of ASD patients cannot be inferred from the present study. Further studies are necessary to clarify if genetic variation of fatty acid oxidation and interaction with environmental factors including diet might account for acyl-carnitine accumulation. In view of the wide clinical features related to ASD we consecutively recruited patients with ASD diagnosis representing an heterogeneous ASD population: further studies are required to understand possible genetic and behavioral correlates of metabolic subtypes of ASD. One limitation of the present study is the lack of inclusion of a neurodevelopmental delay group to understand the performances of the algorithms for ASD vs. other developmental disabilities. Moreover, our study has been developed in a clinical sample. Similarly, classifiers have been applied to identify biomarkers of neurological and psychiatric diseases in clinical cohorts (28, 59). However, it has been recently highlighted that machine learning models should be adjusted to the epidemiological prevalence in the general population (60). Larger-scale studies or population analyses are therefore needed to assess performances in real life cohorts considering the actual prevalence rate. This will require resources for large-scale collaborative efforts worldwide (61).

## Conclusion

The present study supports early recognition of a distinctive metabolic profile in DBS whose distinguishing features suggest a reduced flux through the mitochondrial fatty acid β-oxidation pathway and provides insight into concealed molecular mechanisms determining ASD. The results show higher classification performances in children with ASD younger than 5 years old suggesting a potential complementary and supportive ability to improve diagnosis at earlier stages of ASD development. The applied non-invasive methodology on DBS traditionally used for newborn screening is appropriate to evaluate metabolic profile changes across development. The present findings yield the evidence that metabolic biomarkers that identify subset of patients with ASD are helpful. Considering the heterogeneity of ASD, metabolic profiling may support the identification of phenotypes enabling individualized therapeutic approaches in children at risk of developing the disease.

## Data Availability Statement

The raw data supporting the conclusions of this manuscript will be made available by the authors, without undue reservation, to any qualified researcher.

## Author Contributions

RB and SA conceived the project with contributions by MM, AP, and AF. MM and ADP analyzed the clinical samples. MM, ADP and GT acquired the data and performed data analyses. RB, MG, FM, AGF, GR carried out participants' recruitment and clinical data analysis. SA, AP, AF performed statistical data analysis and computational analyses. RB and SA wrote the paper. RR, JB, RF performed critical revision of the manuscript for intellectual contents. All Authors read and approved the final manuscript.

## MIMIC-Autism Group

List of coauthors included in MIMIC (Metabolism-Immunity-Metals Integrated Concepts), Autism project: a multilevel approach to understand and cure Autism Spectrum Disorders.

### Conflict of Interest Statement

The authors declare that the research was conducted in the absence of any commercial or financial relationships that could be construed as a potential conflict of interest.

## Abbreviations

ASD: autism spectrum disorders
IEM: inborn errors of metabolism
ESI-MS/MS: electrospray ionization-tandem mass spectrometry
DBS: dried blood spots
TD: typically developing
FAO: fatty acid β-oxidation
ADI-R: Autism Diagnostic Interview-Revised
ADOS: Autism Diagnostic Observation Schedule
CSS: Calibrated Severity Score
DD: developmental delay
ID: intellectual disability
PPV: Positive Predictive Value
NPV: Negative Predictive Value
DOR: Diagnostic Odds Ratio
RF: Random Forest
SVM: Support Vector Machine
LM: Linear Regression
PART: Recursive Partition Tree
SCAD: short chain acyl-CoA dehydrogenase
MCAD: medium chain acyl-CoA dehydrogenase
VLCAD: very long chain acyl-CoA dehydrogenase
LCHAD: long chain 3-hydroxyacyl-CoA dehydrogenase
PA: propionic academia
PPA: propionic acid
NSC: neural stem cells
CPT: carnitine palmitoyl transferase.
