# Supplementary material for: A Subset of Patients With Autism Spectrum Disorders Show a Distinctive Metabolic Profile by Dried Blood Spot Analyses
Source: Front Psychiatry. 2018 Dec 7;9:636. doi: 10.3389/fpsyt.2018.00636 (PMC6292950; doi:10.3389/fpsyt.2018.00636)

**Supplementary Materials**

Title: A subset of patients with Autism Spectrum Disorders show a distinctive metabolic profile by dried blood spot analyses

Authors: Rita Barone^1-2§*^, Salvatore Alaimo^3§^, Marianna Messina^2^, Alfredo Pulvirenti^3^, Jean Bastin^4-5^, MIMIC-Autism group, Alfredo Ferro^2^, Richard E. Frye^6-7^, Renata Rizzo^1^

Affiliations: ^1^Child Neurology and Psychiatry; ^2^Referral Centre for Inherited Metabolic Disorders ^3^Bioinformatics Unit; - Department of Clinical and Experimental Medicine - University of Catania, Catania, Italy. ^4^Sorbonne Paris Cité, Faculté des Sciences Fondamentales et Biomédicales, Université Paris Descartes, Paris, France. ^5^INSERM, UMR-S 1124, Paris, France. ^6^University of Arizona College of Medicine, Phoenix, AZ, United States.^7^Phoenix Children's Hospital, Phoenix, AZ, United States.

Correspondence: Dr. Rita Barone

[rbarone@unict.it](mailto:rbarone@unict.it)

**Methods**

**Naïve Bayes Classifier**

A naive Bayes classifier is a simple probabilistic method which applies Bayes' theorem with a naive independence assumption between the features of the model in order to classify samples. Let $X=\left( x_{i},\ldots, x_{n} \right)$ be a vector of $n$ features corresponding to a patient to be classified and let $C_{k}$ be a label used to classify the sample (i.e. ASD, TD). The algorithm computes the probability $p\left( C_{k}|x_{i},\ldots, x_{n} \right)$ for each $C_{k}$, and assigns to $X$ the class, which has the highest probability value. Using Bayes’ theorem, $p\left( C_{k}|x_{i},\ldots, x_{n} \right)$ is decomposed as:

$$p\left( C_{k}|x_{i},\ldots, x_{n} \right)=\frac{p\left( C_{k} \right)p\left( x_{i},\ldots, x_{n}|C_{k} \right)}{p\left( x_{i},\ldots, x_{n} \right)}.$$

Since $p\left( x_{i},\ldots, x_{n} \right)$ is always the same for each assignment of $C_{k}$, the above expression can be approximated in:

$$p\left( C_{k}|x_{i},\ldots, x_{n} \right)\propto p\left( C_{k} \right)p\left( x_{i},\ldots, x_{n}|C_{k} \right)=p\left( C_{k},x_{i},\ldots, x_{n} \right).$$

By employing the chain rule for repeated applications of the definition of conditional probability, and the naïve conditional independence assumption, the above probability can be further simplified in:

$$p\left( C_{k}|x_{i},\ldots, x_{n} \right)\propto p\left( C_{k} \right)\prod_{i=1}^{n} p\left( x_{i}|C_{k} \right).$$

Starting from a set of training samples, such probabilities are computed, and new samples can be classified.

**Other classification models**

We compared the model with C-tree, Random Forest (RF), Support Vector Machine (SVM), Linear Regression (LM), and Recursive Partition Tree (PART).^1-5^

C-tree is a methodology that, starting from input data, builds a tree structure in which each internal node represents a test on a specific subject attribute, and each branch represents one of the possible test outcomes. Each branch can lead to additional tests on other attributes, or to assign a category to the subject (i.e. affected or healthy). The C-tree algorithm also employs a conditional inference procedure based on test-statistic in order to select the most significant variables for the classification.

RF is a technique that, starting from input data, builds a set of decision trees to classify such samples. The result of the classification of a new subject is given by the class that most trained decision trees return.

SVM is a classification model that, given a set of points in an n-dimensional space, divides them into two classes by building a hyperplane, which best separates the two categories. In this way the space will be divided into two subspaces, and to each of them a specific class is assigned. A new sample is, therefore, classified on the basis of its position in space.

Linear regression is a tool that, starting from values measured on some subjects, find the parameters of a function $f\left( x \right)=\beta_{0}+\sum\left( \beta_{i}\cdot x_{i} \right)$, where $x_{i}$ is the value of the *i*-th measured characteristic on a subject, $f\left( x \right)$ is its class, and $\beta_{i}$ is a weight associated to the *i*-th feature of the subject. The aim of the linear regression is to find all $\beta_{i}$ values so that the classification error is minimized.

Finally, PART is a classification method based on decision trees, which divides the population of subjects into two subpopulations based on a specific characteristic. This breakdown is recursively repeated to each subpopulation until all subpopulations contains samples from a homogeneous class.

**References**

1. Hothorn T, Hornik K, Zeileis A. Unbiased Recursive Partitioning: A Conditional Inference Framework. J Comput Graph Stat. 2006; 15(3):651–74.
2. Strobl C, Malley J, Tutz G. An introduction to recursive partitioning: rationale, application, and characteristics of classification and regression trees, bagging, and random forests. Psychol Methods. 2009 ;14(4):323-48.
3. Cortes C, Vapnik V. Support-vector networks. Machine learning 1995;20: 273-97.
4. Chambers JM. Linear models. In: JM Chambers and TJ Hastie eds, Statistical Models in S. Wadsworth & Brooks/Cole, 1992.
5. Breiman L., Friedman JH, Olshen RA, Stone CJ. Classification and Regression Trees. Wadsworth, 1984.

**Tables S1**: performances of classification algorithms used by randomizing class labels (ASD and TD) 1000 times in all participants (A), participants aged ≤ 5 years (B) and > 5 years (C) demonstrating a fall of classification performance. The results show that the quality of the classification in such a case is even lower than expected values in the case of a random classifier model.

Compared classification algorithms: Naïve Bayes, C-tree, Random Forest (RF), Support Vector Machine (SVM), Linear Regression Model (LM), and Recursive Partition Tree (PART). For each classifier accuracy, true positive rate (TPR), true negative rate (TNR), false positive rate (FPR), false negative rate (FNR), of the model and diagnostic odds ratio (DOR) are shown. All the measures are reported together with the bounds of the 95% CI.

1. **All participants**

| **Algorithm** | **accuracy** | **TPR** | **TNR** | **FPR** | **FNR** | **DOR** |
| --- | --- | --- | --- | --- | --- | --- |
| **Naive Bayes** | **0.6775 [0.6735; 0.6815]** | **0.7332 [0.7267; 0.7397]** | **0.6345 [0.6287; 0.6404]** | **0.3655 [0.3596; 0.3713]** | **0.2668 [0.2603; 0.2733]** | **6.7823 [6.3956; 7.1690]** |
| C-Tee | 0.5715 [0.5674; 0.5756] | 0.6715 [0.6590; 0.6841] | 0.4942 [0.4824; 0.5060] | 0.5058 [0.4940; 0.5176] | 0.3285 [0.3159; 0.3410] | 2.7437 [2.6171; 2.8703] |
| RF | 0.6379 [0.6339; 0.6418] | 0.7296 [0.7229; 0.7364] | 0.567 [0.5608; 0.5731] | 0.433 [0.4269; 0.4392] | 0.2704 [0.2636; 0.2771] | 4.8079 [4.5668; 5.0490] |
| SVM Linear | 0.6541 [0.6500; 0.6581] | 0.7319 [0.7250; 0.7388] | 0.5939 [0.5875; 0.6003] | 0.4061 [0.3997; 0.4125] | 0.2681 [0.2612; 0.2750] | 5.5428 [5.2650; 5.8207] |
| LM | 0.6471 [0.6430; 0.6511] | 0.6324 [0.6250; 0.6397] | 0.6585 [0.6523; 0.6646] | 0.3415 [0.3354; 0.3477] | 0.3676 [0.3603; 0.3750] | 4.4804 [4.2328; 4.7280] |
| PART | 0.5929 [0.5883; 0.5975] | 0.6281 [0.6196; 0.6365] | 0.5657 [0.5576; 0.5738] | 0.4343 [0.4262; 0.4424] | 0.3719 [0.3635; 0.3804] | 2.998 [2.8474; 3.1486] |

1. **Age ≤ 5 years**

| **Algorithm** | **accuracy** | **TPR** | **TNR** | **FPR** | **FNR** | **DOR** |
| --- | --- | --- | --- | --- | --- | --- |
| **Naive Bayes** | **0.7222 [0.7161; 0.7283]** | **0.7237 [0.7137; 0.7337]** | **0.7209 [0.7125; 0.7293]** | **0.2791 [0.2707; 0.2875]** | **0.2763 [0.2663; 0.2863]** | **10.1235 [ 9.4725; 10.7746]** |
| C-Tee | 0.5448 [0.5392; 0.5504] | 0.759 [0.7441; 0.7739] | 0.3574 [0.3399; 0.3749] | 0.6426 [0.6251; 0.6601] | 0.241 [0.2261; 0.2559] | 2.8163 [2.6411; 2.9914] |
| RF | 0.6773 [0.6709; 0.6838] | 0.827 [0.8187; 0.8353] | 0.5464 [0.5358; 0.5569] | 0.4536 [0.4431; 0.4642] | 0.173 [0.1647; 0.1813] | 7.1375 [6.5862; 7.6888] |
| SVM Linear | 0.7049 [0.6983; 0.7115] | 0.8017 [0.7925; 0.8109] | 0.6202 [0.6105; 0.6300] | 0.3797 [0.3700; 0.3895] | 0.1983 [0.1891; 0.2075] | 8.1844 [7.6207; 8.7481] |
| SVM Radial Basis | 0.6965 [0.6908; 0.7022] | 0.8441 [0.8361; 0.8522] | 0.5672 [0.5582; 0.5763] | 0.4328 [0.4237; 0.4418] | 0.1559 [0.1478; 0.1639] | 7.4587 [6.9656; 7.9518] |
| LM | 0.6729 [0.6663; 0.6796] | 0.6567 [0.6450; 0.6684] | 0.6871 [0.6771; 0.6972] | 0.3129 [0.3028; 0.3229] | 0.3433 [0.3316; 0.3550] | 7.1923 [6.6648; 7.7198] |

1. **Age >5 years**

| **Algorithm** | **accuracy** | **TPR** | **TNR** | **FPR** | **FNR** | **DOR** |
| --- | --- | --- | --- | --- | --- | --- |
| **Naive Bayes** | **0.6149 [0.6091; 0.6206]** | **0.6757 [0.6665; 0.6849]** | **0.5692 [0.5610; 0.5775]** | **0.4308 [0.4225; 0.4390]** | **0.3243 [0.3151; 0.3335]** | **4.2905 [4.0193; 4.5616]** |
| C-Tee | 0.5298 [0.5258; 0.5337] | 0.5071 [0.4867; 0.5275] | 0.5468 [0.5290; 0.5645] | 0.4532 [0.4355; 0.4710] | 0.4929 [0.4725; 0.5133] | 1.6693 [1.5826; 1.7559] |
| RF | 0.552 [0.5462; 0.5577] | 0.5881 [0.5780; 0.5982] | 0.5248 [0.5157; 0.5339] | 0.4752 [0.4661; 0.4843] | 0.4119 [0.4018; 0.4220] | 2.3622 [2.2069; 2.5175] |
| SVM Linear | 0.6116 [0.6060; 0.6173] | 0.5878 [0.5780; 0.5976] | 0.6295 [0.6208; 0.6382] | 0.3705 [0.3618; 0.3792] | 0.4122 [0.4024; 0.4220] | 3.9369 [3.6500; 4.2238] |
| LM | 0.603 [0.5972; 0.6087] | 0.6039 [0.5939; 0.6139] | 0.6022 [0.5933; 0.6112] | 0.3978 [0.3888; 0.4067] | 0.3961 [0.3861; 0.4061] | 3.7105 [3.4456; 3.9755] |
| PART | 0.5323 [0.5262; 0.5383] | 0.5264 [0.5143; 0.5386] | 0.5367 [0.5260; 0.5474] | 0.4633 [0.4526; 0.4740] | 0.4736 [0.4614; 0.4857] | 2.003 [1.8714; 2.1347] |

**Figure S1**: volcano plot showing the distribution of log-fold-changes versus statistical significance (p-value) of the metabolites in our study.


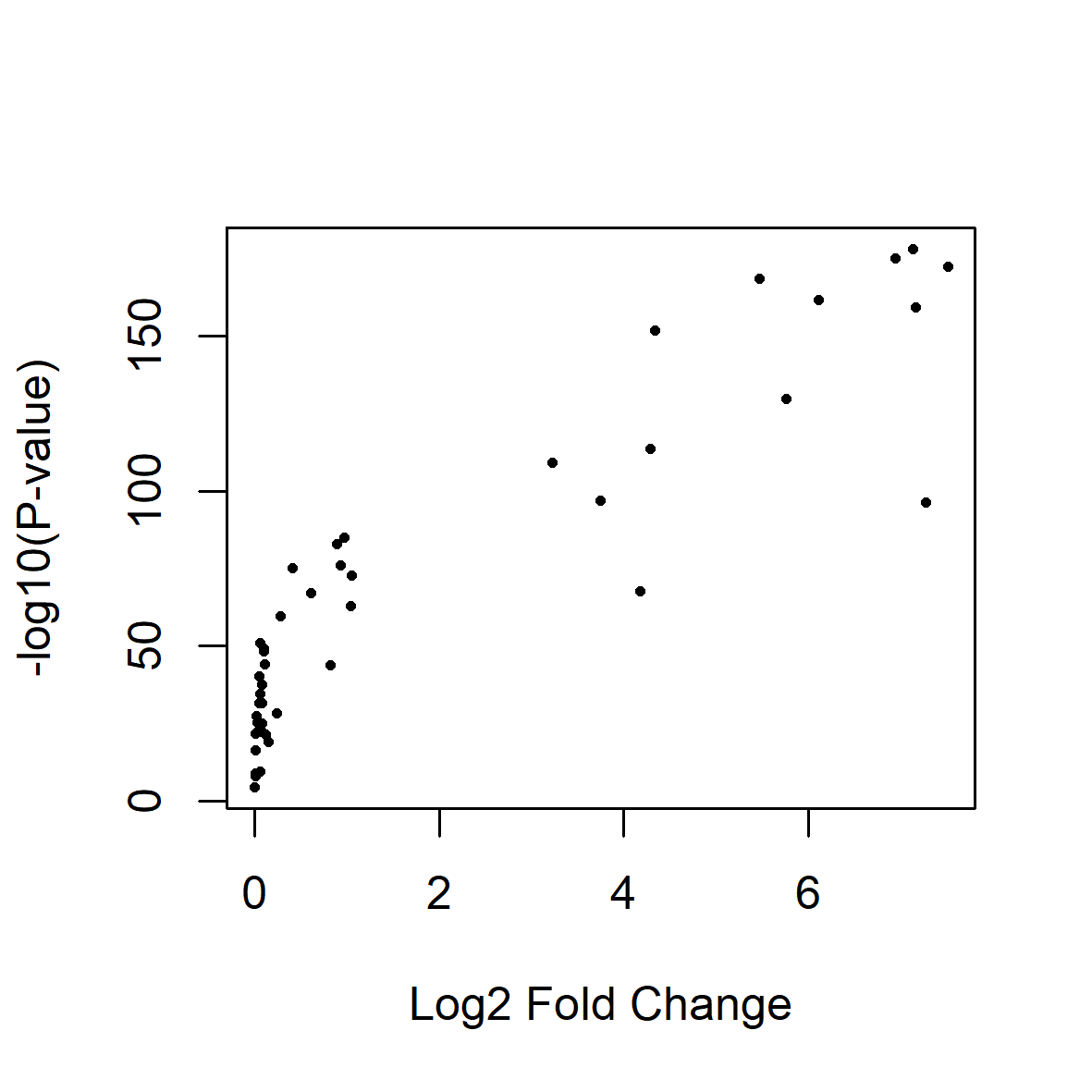


**Figure S2-S4**: swarm plot of the concentrations for the nine changed metabolites in all participants (**Fig.S2**), in participants aged ≤5 years **Fig. S3**) and aged > 5 years (**Fig.S4**) divided by group (ASD and TD). For C12, C14.2, and C16.1 "stepping" of the values are equally represented in the two groups and depend on very low concentration of these metabolites and limited resolution of ESI-MS/MS at such low levels.

**Figure S2**


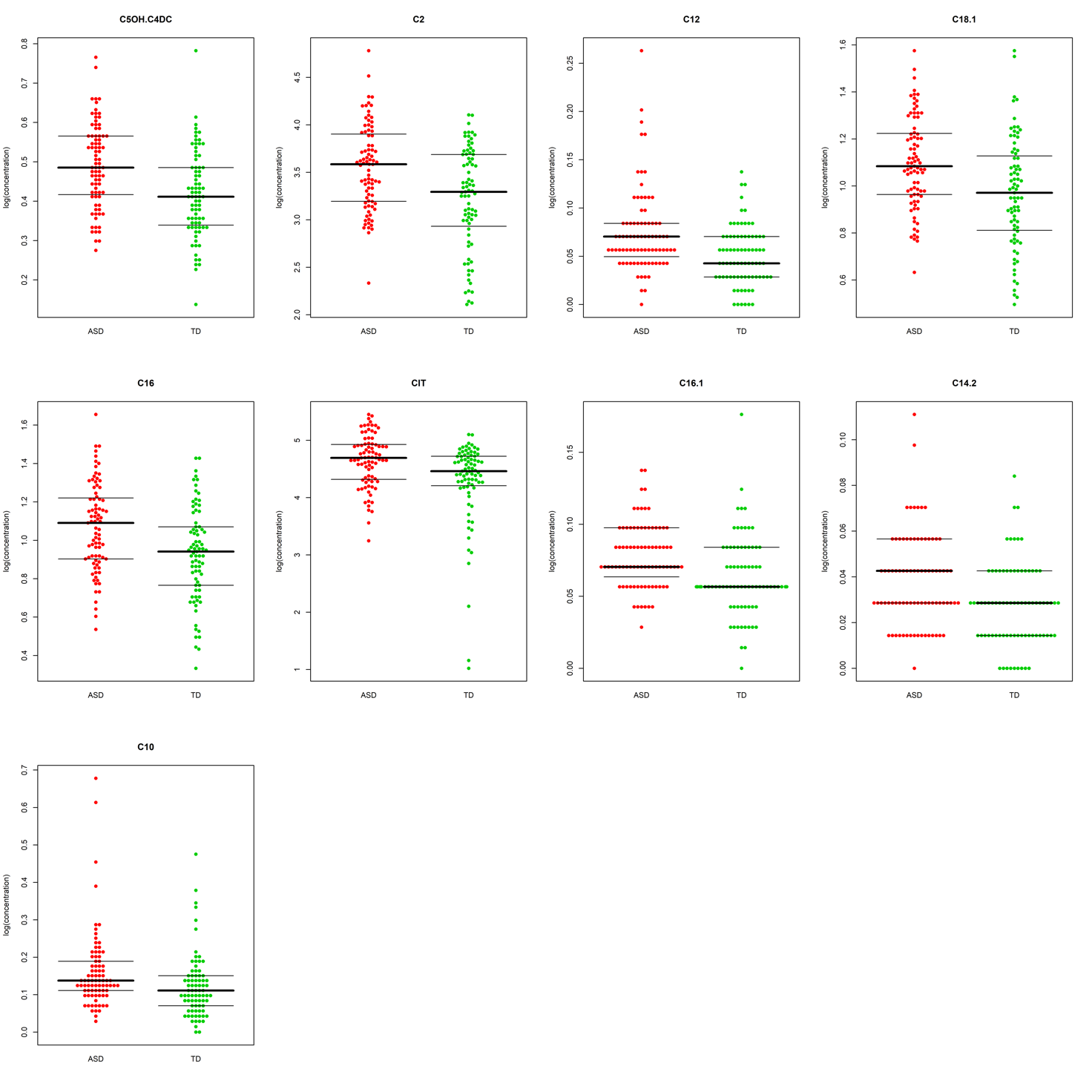


**Figure S3**


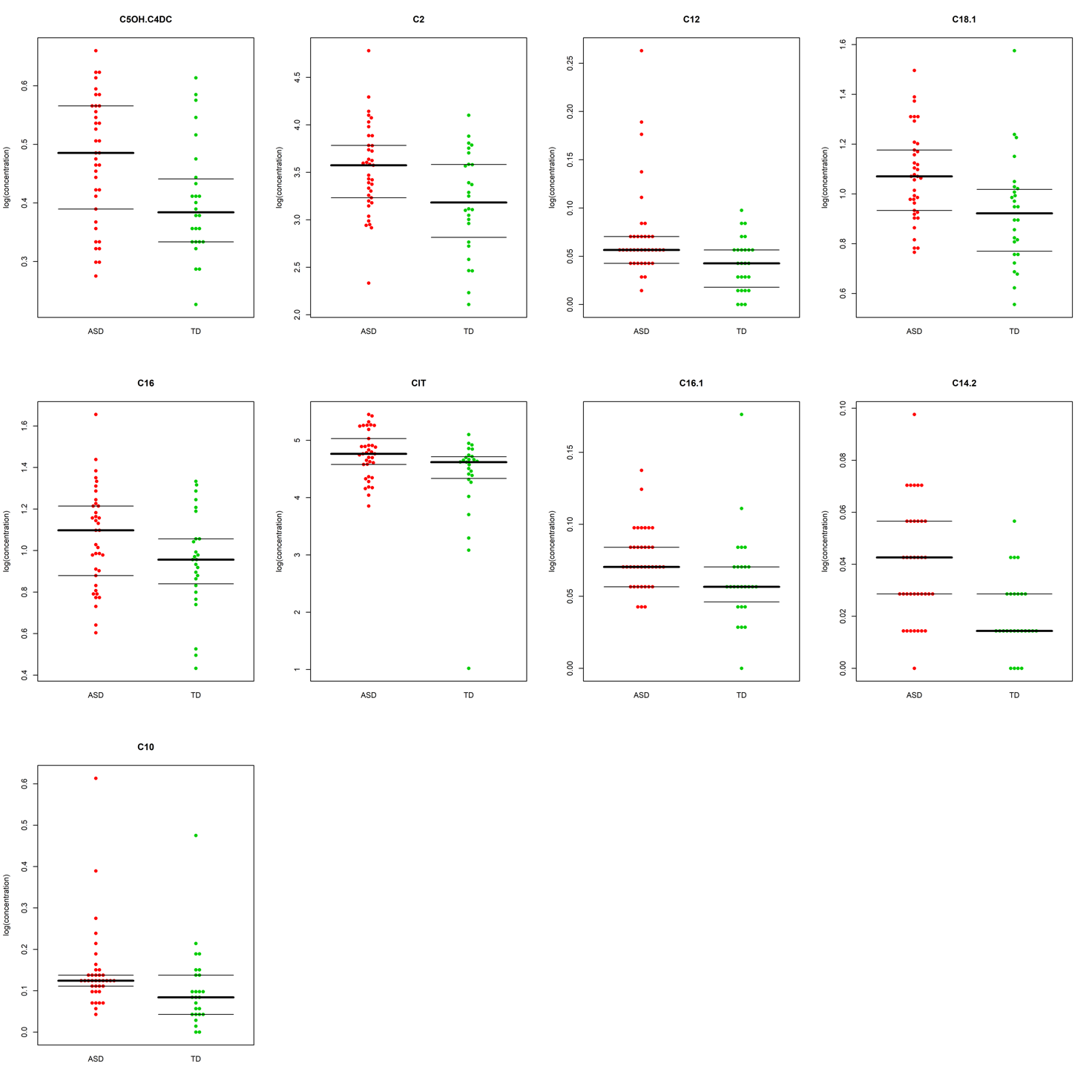


**Figure S4**


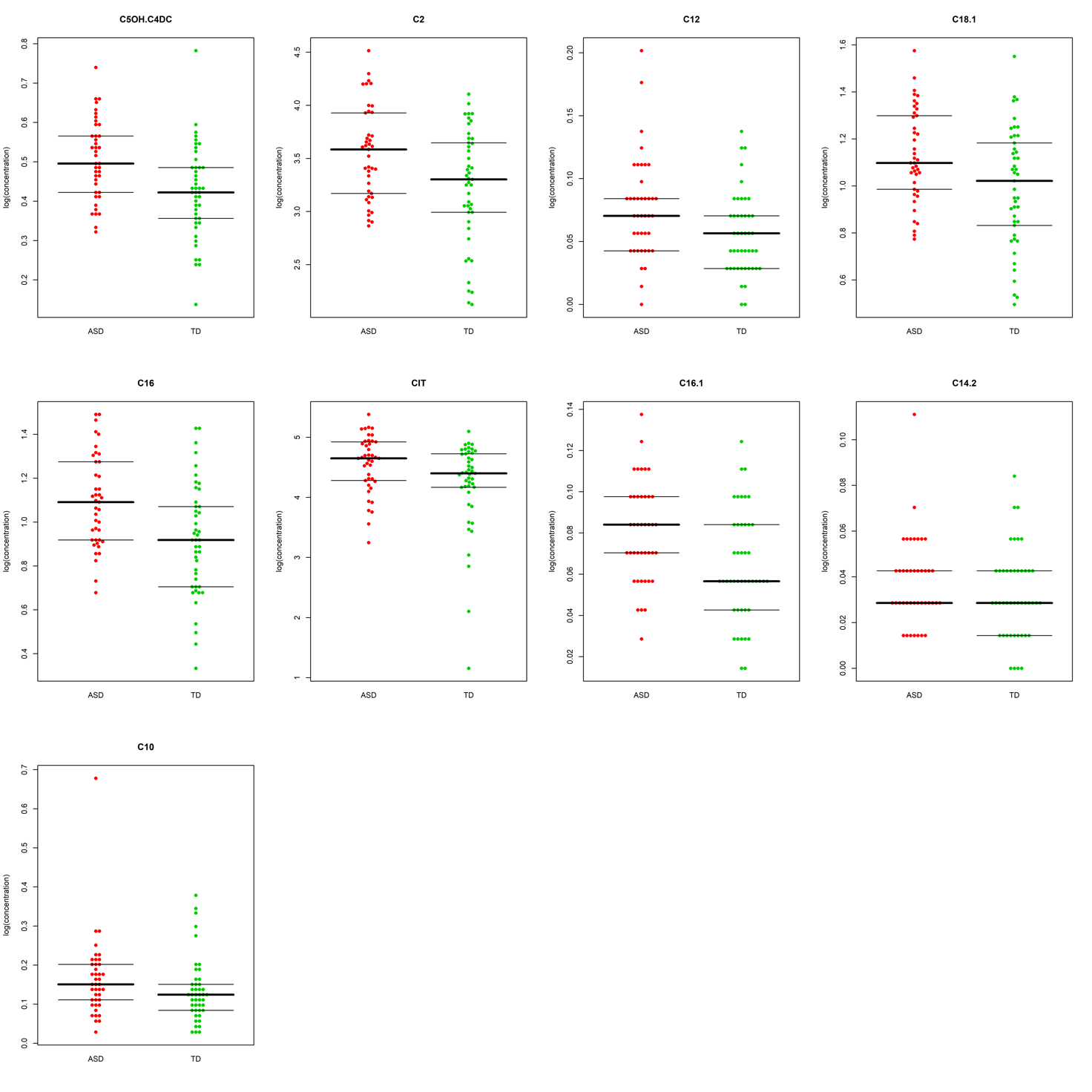

Supplement: Supplementary file 1 [file Data_Sheet_1.docx]
